# Machine learning for screening and predicting the risk of anti-MDA5 antibody in juvenile dermatomyositis children

**DOI:** 10.3389/fimmu.2022.940802

**Published:** 2023-01-10

**Authors:** Yuan Xue, Junmei Zhang, Chao Li, Xuanyi Liu, Weiying Kuang, Jianghong Deng, Jiang Wang, Xiaohua Tan, Shipeng Li, Caifeng Li

**Affiliations:** Department of Rheumatology, Beijing Children's Hospital, Capital Medical Universtity, National Centre for Children's Health, Beijing, China

**Keywords:** antiMDA5, pediatric, juvenile dermatomyositis, antibody, machine learning

## Abstract

**Objective:**

The anti-MDA5 (anti-melanoma differentiation associated gene 5) antibody is often associated with a poor prognosis in juvenile dermatomyositis (JDM) patients. In many developing countries, there is limited ability to access myositis- specific antibodies due to financial and technological issues, especially in remote regions. This study was performed to develop a prediction model for screening anti-MDA5 antibodies in JDM patients with commonly available clinical findings.

**Methods:**

A cross-sectional study was undertaken with 152 patients enrolled from the inpatient wards of Beijing Children’s Hospital between June 2018 and September 2021. Stepwise logistic regression, least absolute shrinkage and selection operator (LASSO) regression, and the random forest (RF) method were used to fit the model. Model discrimination, calibration, and decision curve analysis were performed for validation.

**Results:**

The final prediction model included eight clinical variables (gender, fever, alopecia, periungual telangiectasia, digital ulcer, interstitial lung disease, arthritis/arthralgia, and Gottron sign) and four auxiliary results (WBC, CK, CKMB, and ALB). An anti-MDA5 antibody risk probability–predictive nomogram was established with an AUC of 0.975 predicted by the random forest algorithm. The model was internally validated by Harrell’s concordance index (0.904), the Brier score (0.052), and a 500 bootstrapped satisfactory calibration curve. According to the net benefit and predicted probability thresholds of decision curve analysis, the established model showed a significantly higher net benefit than the traditional logistic regression model.

**Conclusion:**

We developed a prediction model using routine clinical assessments to screen for JDM patients likely to be anti-MDA5 positive. This new tool may effectively predict the detection of anti-MDA5 in these patients using a non-invasive and efficient way.

## Background

Juvenile dermatomyositis (JDM) is an autoimmune disease of childhood affecting 1.9 patients per million children in the United Kingdom (1)and 2.4–4.1 patients per million children in the USA (2). The mortality rate of JDM in developed countries is currently estimated at 2%–3% (3).

JDM has been reported to be of significant heterogeneity: the clinical symptoms are widely diverse including muscle and skin involvement, interstitial lung disease (ILD), arthritis, and cardiac inflammation. Among them, anti-MDA5 (anti-melanoma differentiation associated gene 5) antibodies are related to a poor prognosis (4). It is of great significance to identify such subtype at an early stage in an efficient, economical, and non-invasive way.

**Figure 1:**
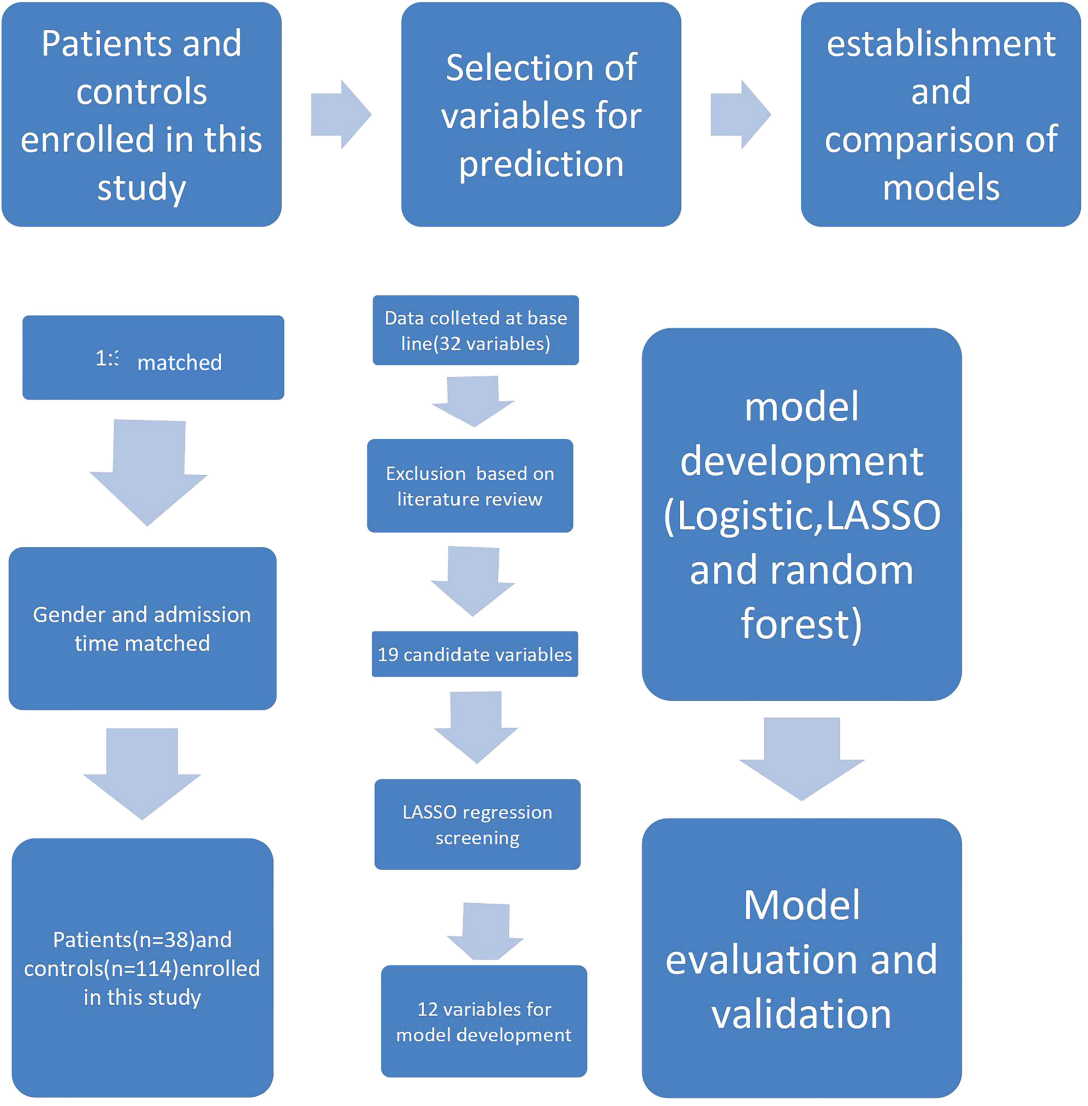
Flow chart showing the study design.

In this study, we adopted stepwise logistic regression, least absolute shrinkage and selection operator (LASSO) logistic regression, and LASSO regression combined with the random forest method to develop a suitable model for screening and predicting the risk of anti-MDA5 antibodies in JDM patients. The selected predictors are representative and covered diverse and typical organs involved in JDM, and, together, they presented with a superior predictive performance. The technological process of the study is shown in **Figure 1**.

## Patients and methods

The methods used in our article follow the Transparent Reporting of a Multivariable Prediction Model for Individual Prognosis or Diagnosis (TRIPOD) statement (5).

### Participants

A total of 152 patients with JDM from the inpatient wards of Beijing Children’s Hospital between June 2018 and September 2021 were enrolled in the cohort in our study. Among them, 38 patients were positive for anti-MDA5 antibodies. These patients were used for a case–control study. The control group was entry time and race-matched who were negative for anti-MDA5 antibodies at the ratio of 1:3 as the control group (114 patients). All patients with JDM fulfilled Bohan and Peter criteria or the 2017 EULAR/ACR classification criterion for dermatomyositis (6–8) (**Supplementary Table 1**). The medical records of all patients were retrospectively reviewed. Patients with juvenile polymyositis (JPM) had been excluded to minimize the interference as JPM was identified as a distinct subtype based on a different pathological mechanism from JDM. Ethics approval and informed patient consent from patients and their guardians had been obtained and collected. The myositis antibodies were tested by the Western blot method. The antibody distribution of the 152 patients is shown in **Supplementary Table 2**.

### Patient assessment and data collection

The time of recruitment was defined as the time of the JDM diagnosis confirmed by our research group (initial diagnosis). We collected data from the initial diagnostic evaluation, including the medical history, disease characteristics, and laboratory evaluations. Two investigators (Y.Xue and J.Zhang) blinded to the standardized data collection forms ascertained the outcomes.

### Development of the prediction model

The least absolute shrinkage and selection operator (LASSO) logistic model was performed to prevent overfitting on the basis of stepwise logistic regression to select the predictive variables from the 19 potential candidate variables preprocessed based on literature review and expert opinion (one chief physician and three deputy chief physicians who worked in our medical center whose outpatient department made over 100 diagnoses of JDM per year. Each variable was measured by a rheumatologist separately to determine stability and the predictive value. Literature review was also performed to identify variables with sufficient evidence as predictors in the model, as shown in **Supplementary Method**). For variable selection and regularization to maximize the prediction accuracy and interpretability, LASSO regression tends to be suitable for data with high multicollinearity (9, 10).

As a machine learning method with the prominent advantages of less restrictions on variable conditions (11) and higher sensitivity and specificity than decision trees, we chose the random forest (RF) algorithm to predict continuous variables and obtain predictions without obvious deviations (12) as a suitable prediction method in this study.

### Selection of predictor variables

In our study, we identified 152 patients with JDM and subgrouped them according to the expression of anti-MDA5 antibodies. The characteristics of patients are shown in **Table 1**. We selected 19 variables including symptoms frequently found in the JDM and the expression features of myositis antibodies and then included these original variables in analysis. The 19 variables were included in the analysis with the following details:

1. Frequently detected and available in JDM
2. No large-scale missing data
3. Variables with similar significance were combined, such as arthritis and arthralgia, for further analysis.

**Table 1:** 19 candidate variables including the demographic and clinical characteristics of the participants analyzed for the risk of anti-MDA5 (anti-melanoma differentiation associated gene 5).

Variables	Anti-MDA5 positive (n=38)	Anti-MDA5 negative (n=114)	P- value
Age (Y)	6.78 ± 3.49	8.19 ± 3.28	0.191
Gender (F, %)	60.5	51.8	0.017*
Symptoms (%)			
Fever	55.3	32.5	0.012*
Alopecia	10.5	1.8	0.017*
Periungual telangiectasia	44.7	21.1	0.005*
Digital ulcer	18.4	4.4	0.006*
Arthritis/arthralgia	59.7	28.9	0.001*
Gottron sign	92.1	74.6	0.022*
Interstitial lung disease (ILD)	78.9	34.2	<0.0001*
Cough	34.2	23.7	0.203
Dyspnea	2.6	2.6	1.000
WBC (X10^9/L)	6.39 ± 3.67	8.71 ± 3.60	<0.0001*
ESR (mm/h)	12.00 (5.00,27.00)	6.00 (2.00,16.25)	0.003*
ALT (U/L)	38.80 (17.00,70.60)	33.30 (18.78,61.00)	0.567
AST (U/L)	48.30 (29.00,105.30)	50.20 (30.00,99.52)	0.607
ALP (U/L)	109.82 ± 48.21	146.49 ± 87.99	0.134
ALB (g/L)	36.17 ± 7.91	43.13 ± 38.16	0.012*
CK (IU/ml)	21.50 (16.00,47.92)	54.00 (37.00,197.00)	<0.0001*
CKMB (IU/ml)	18.00 (12.00,22.00)	20.80 (12.70,47.20)	0.004*

WBC, white blood cell; ESR, erythrocyte sedimentation rate; ALT, alanine transaminase; AST, aspartate transaminase; ALP, alkaline phosphatase; ALB, albumin; CK, creatine kinase; CKMB, creatine kinase myocardial band.* significant difference.

## Results

**Figure 2:**
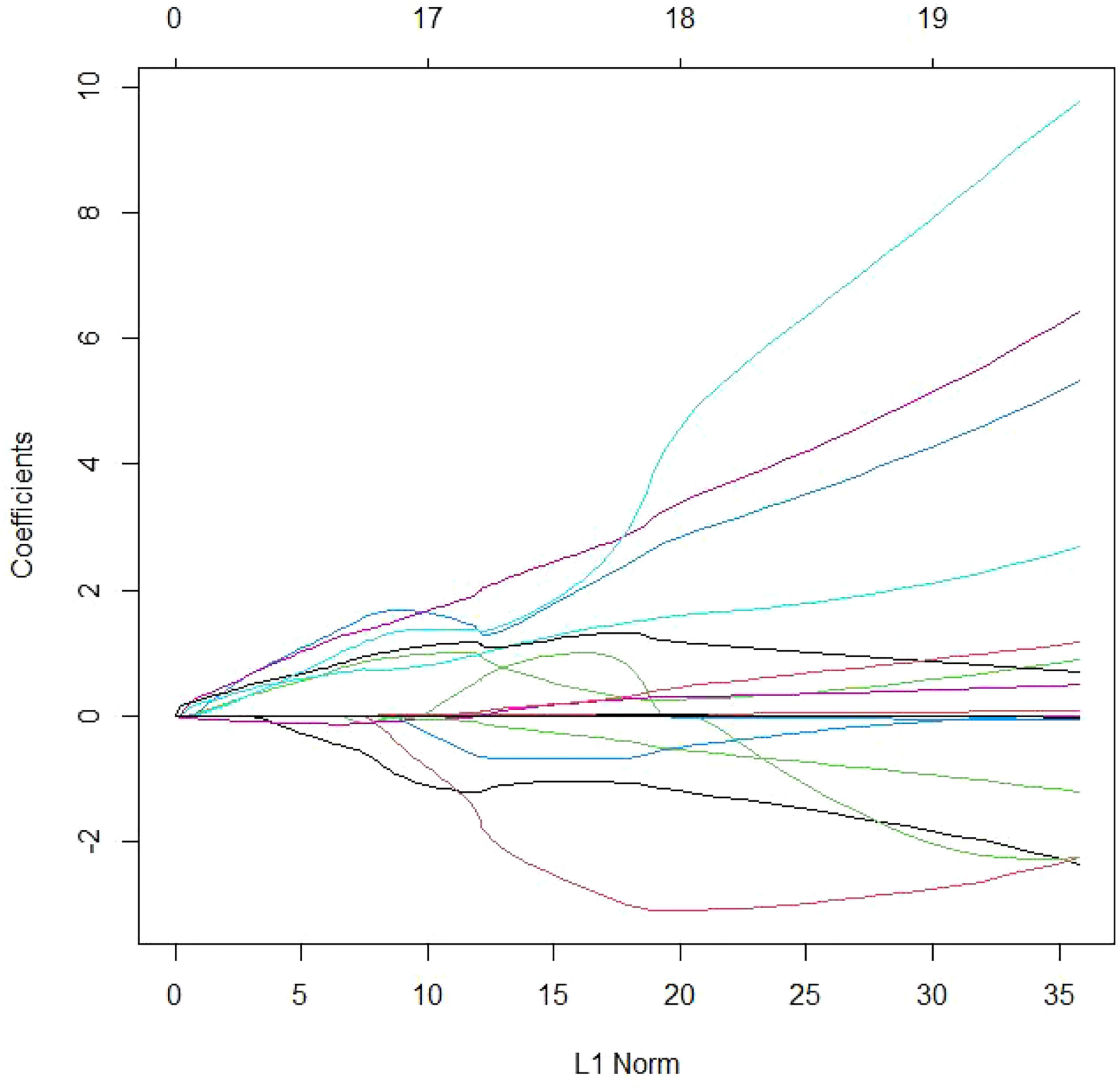
Variable selection using the LASSO logistic Poisson model. LASSO model coefficient profiles of the 19 candidate variables. The logistic regression coefficients are estimated with an upper bound (“L1 norm”) to the sum of the absolute standardized regression coefficients. The L1 norm regularization term typically shrinks many regression coefficients to 0.

**Figure 3:**
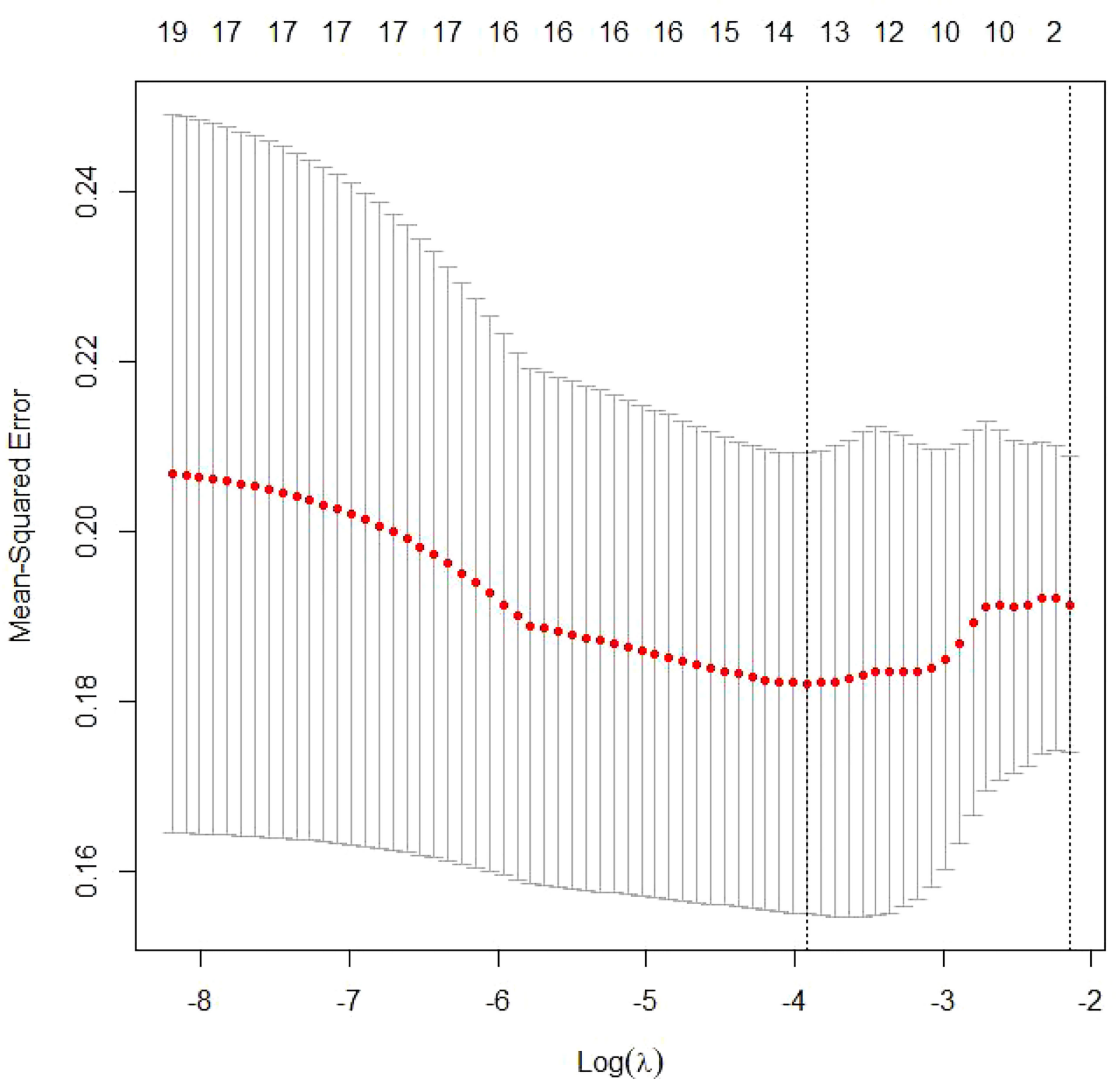
Tuning parameter selection by cross-validation in the LASSO model. The solid vertical lines represent the partial likelihood deviance standard error (SE). The red dotted line indicates the cross-validation curve. The broken vertical lines indicate the optimal values on the basis of the minimum criteria and 1−SE criteria. A λ value of 0.04194893, with a log(λ) value of −4.356293, was chosen according to cross-validation.

There were 12 variables selected using LASSO regression to improve model accuracy and reduce model overfitting (**Figures 2**, **3**) from the 19 candidate variables, which included seven clinical features and five auxiliary examinations. Stepwise logistic regression and RF modeling also confirmed the significance between the variables in different aspects (**Table 2**).

**Table 2:** The estimated coefficients in the stepwise logistic regression and logistic least absolute shrinkage and selection operator regression.

Variable	StepwiseLogistic	LASSO
Gender		-0.044
Fever	1.124	0.002
Alopecia	2.501	0.006
Periungual telangiectasia		0.004
Digital ulcer		0.05
Arthritis/arthralgia	1.077	0.012
Gottron sign	1.614	0.002
ILD		0.005
WBC	-0.218	0.016
CK		0.063
CKMB		-0.053
ALB		-0.003

ILD, interstitial lung disease; WBC, white blood cell; CK, creatine kinase; CKMB, creatine kinase myocardial band; ALB, albumin.

Among the 12 variables, 4 were continuous variables: WBC, CK, CKMB, and ALB.

### Model development

A detection of anti-MDA antibodies was entered as a dependent variable, Y in the logistic regression model was coded as 0 for negative and 1 for positive. The probability of anti-MDA5 positive given the covariates xi was calculated as the following formula:


$$\begin{array}{l} \text{P(Y}=1|\text{xi)}=\text{exp}(\text{β}0+\text{β}1\text{ xi}1+\ldots +\text{βk xik})\\ \;1+\text{exp}(\text{β}0+\text{β}1\text{ xi}1+\ldots +\text{βk xik}) \end{array}$$

where xi=(xi1, xi2,…, xik) are the covariates of the ith observation and include binary and continuous variables. β0 is the intercept, and βj (j=1,…,k) was defined as the coefficient corresponding to the jth covariate.

The logistic LASSO estimator β0, …, βk was the minimizer of negative log likelihood:

∑ n i =1 [-yi (β0+β1 xi1+…+βk xik)+log(1+exp(β0+β1 xi1+…+βk xik))],subject to ∑ k j =1|βj | ≤λ.

The glmnet package of R (version 4.1.3) was applied to obtain the logistic LASSO estimator.

### Random forest

In our study, the variable types of latent factors included both nominal variables and continuous variables with a skewed distribution feature. According to previous studies, RF models for predictions as a suitable ensemble learning algorithm showed the prominent advantages of higher accuracy, sensitivity, and specificity than decision trees (13) and no restrictions on variable conditions (11).

**Figure 4:**
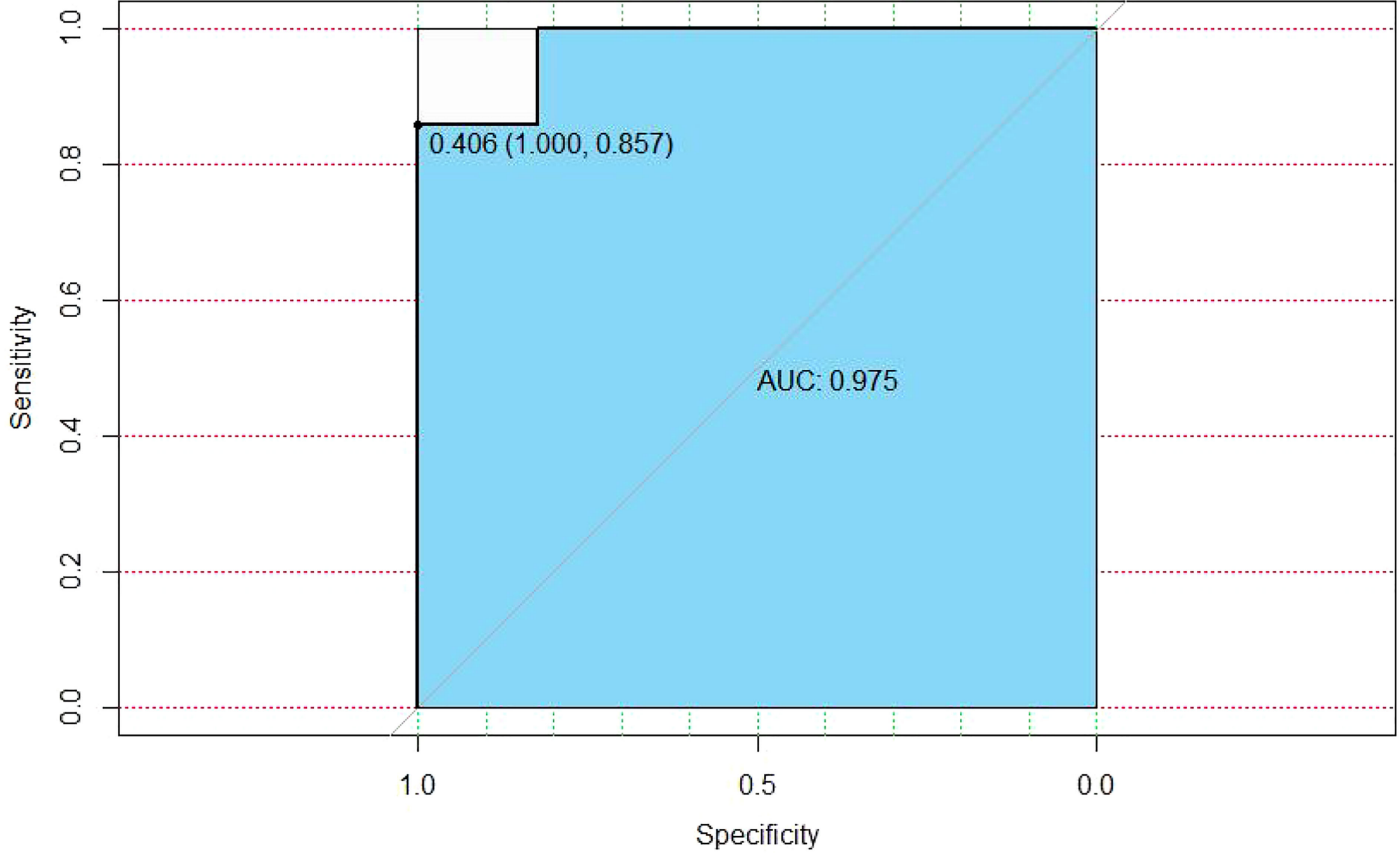
The area under the curve (AUC) of the random forest algorithm for the established model.

We adopted a ratio of the train dataset and prediction dataset with 0.8 to 0.2; the AUC of random forest tended to be 0.975 (**Figure 4**).

### Model performance and internal validation

**Figure 5:**
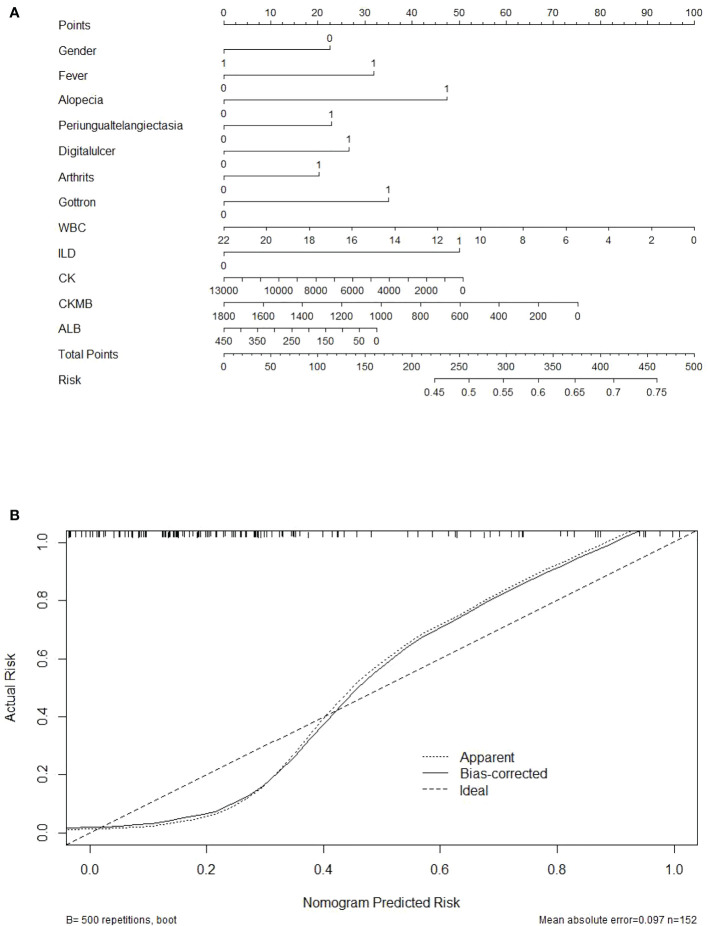
Nomogram and the calibration curve for the anti-MDA5 prediction model. **(A)** Nomogram predicting the probability that a JDM patient will be detection positive for anti-MDA5 antibodies. Points for 12 screened variables can be obtained using a point caliper and then summed to obtain a total score that can be matched with the risk. **(B)** Calibration curve of the prediction model by the actual risk with 500 bootstraps. Broken line represents apparent prediction; solid line represents the performance of the corrected prediction model. A smaller distance between the scatter point and the broken line indicates a better calibration.

The established model showed superiority to the SL regression model in terms of AUC (0.975 vs. 0.729, P<0.001 [both]). The performance of the model for predicting the risk of MDA5 was calculated using the 152 patients with 19 variables. The C-index for the established model was 0.904 with a 500 bootstrap adjustment for optimism to discriminate between patients with JDM patients with MDA5 or without MDA5. The Brier score for the model was 0.052, A calibration plot of 500 bootstrap replications showed the comparison between the predicted risk and the actual risk in the different groups (**Figure 5**).

### Net benefit of the prediction model

**Figure 6:**
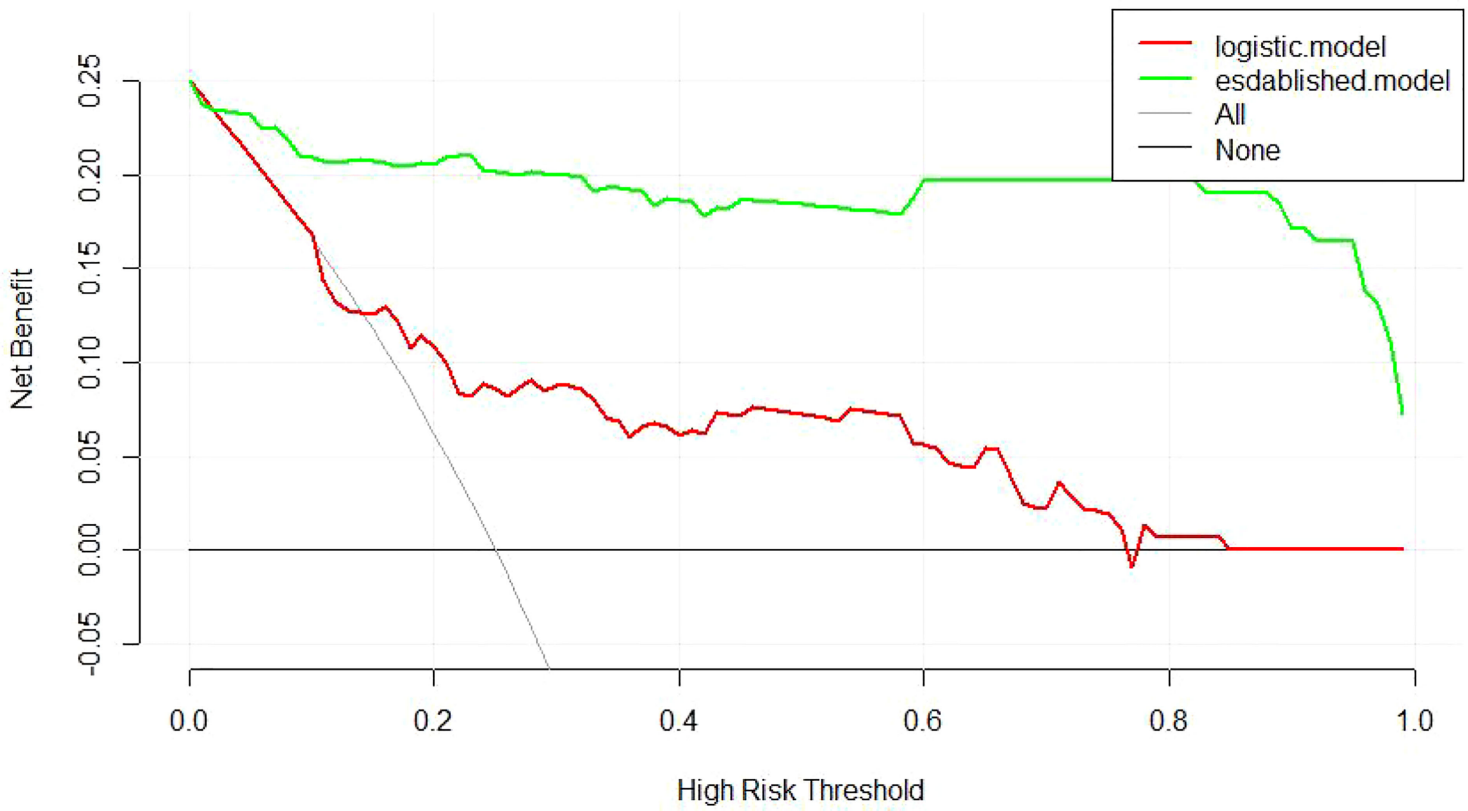
Decision curve analysis of the two models. “None” is the net benefit when it is assumed that none of the JDM patients will have the outcome (anti-MDA5 positive). “All” is the net benefit when it is assumed that all the JDM patients will have the outcome. ‘established’’logistic’ represents the net benefit when JDM patients are screened the predicted risk of anti-MDA5 estimated by different prediction models, respectively. Thestrategy with the highest net benefit at any given threshold is the preferred strategy.

Decision curve analysis was applied to compare the efficiency between different models. A positive net benefit for probability thresholds between 1% and 100% compared with screening as if all of the JDM patients would be detected with anti-MDA5 or screening as if none of the JDM patients were screened in all the two models. As shown in **Figure 6**, the established model presented with a higher net profit than the model of logistic regression, which was in accordance with the result of ROC analysis.

## Discussion

As a developing country, there is limited access due to expense and the lack of available technology for the detection of myositis- related antibodies in some remote areas in China. In this study, we developed an internally validated clinical model to predict the absolute risk of anti-MDA5 positivity in a representative cohort of JDM patients. The model incorporated routine clinical parameters, making it available in regular practice. The established nomogram helped to discriminate between patients who may develop anti-MDA5 antibodies and those who did not with appropriate calibration (e.g., due to expense, the lack of available technology, or other reasons, anti-MDA5 antibodies failed to be detected). Decision curve analysis revealed the clinical utility of the established model over a wide range of probability thresholds with a significant higher net profit than the traditional logistic model, and the model is relevant for pediatric rheumatologists for identifying JDM patients who are at high risk for the detection of anti-MDA5 antibodies.

In our research work, we found that the JDM patients with anti-MDA5 antibodies have an earlier onset age than those without anti-MDA5 (6.78 ± 3.49y vs. 8.19 ± 3.28y, p = 0.017).The patients with anti-MDA5 showed higher rates of fever (55.3% vs. 32.5%, p = 0.012), alopecia (10.5% vs. 1.8%, p = 0.017), periungual telangiectasia (44.7% vs. 21.1%, p = 0.005), Gottron’s sign (92.1% vs. 74.6%, p = 0.022), ILD (78.9% vs. 34.2%, p< 0.00001), and arthritis (59.7% vs. 28.9%, p = 0.001). The results of laboratory tests showed that the JDM patients with anti-MDA5 antibodies have lower WBC counts in peripheral blood (6.39 ± 3.67 X 10^9/L vs. 8.71 ± 3.60X 10^9/L, p< 0.0001), decreased CK [21.5(16.00, 47.92) vs. 54.00(37.00, 197.00), p< 0.0001], and CKMB [18.00 (12.00, 22.00) vs. 20.80 (12.70, 47.20), p = 0.004], albumin in serum (36.17 ± 7.91 vs 43.13 ± 38.16, p = 0.012) and elevated ESR[12.00(5.00,27.00) vs 6.00(2.00,16.25)].The results indicated that cutaneous, articular, and pulmonary manifestations were more frequently found in patients with anti-MDA5 antibodies while muscular involvement is milder, which was generally consistent with previous reports (14–18). Among DM- associated autoantibodies, the anti-MDA5 antibody is the one most associated with ILD, some researchers assumed that the infection of the skin and lung epithelium by certain viruses may upregulate the expression of MDA5 in the infected tissues, which may lead to the high relevance with skin rash and ILD in anti-MDA5 patients (19).The elevated ESR and decreased serum albumin (while ALT and AST both failed to show significant difference while compared between anti-MDA5- positive and anti-MDA5- negative groups) may indicate an inflammatory response in JDM patients with anti-MDA5, which is consistent with previous reports declaring that the ferritin level was higher in anti-MDA5 antibody- positive patients (20, 21). Further research is required to clarify this.

The prediction model we developed in this study has several advantages over other available models currently for screening anti-MDA5 antibodies. First, compared with previous cross-sectional or case–control studies (4, 15, 22), our cohort permitted the analysis of identified predictors to screen anti-MDA5 antibodies in JDM patients by establishing an evaluation system rather than identifying and concentrating on isolated risk factors. Second, the model was established using frequently seen clinical variables, which means that it can be applied well in clinical settings. Third, the model showed robust clinical stability and satisfactory predictive effectiveness in internal validation, which lay a reference foundation for external validation.

In our work, the coefficients estimated by SL regression tended to be quite large compared to those estimated using LASSO regression. The unbalanced distribution of some covariates may lead to the inflation of the estimated coefficients and the inferior predictive performance of SL regression. Meanwhile, LASSO regression shrinks such coefficients in order to avoid the inflation of the estimated coefficients resulting in superior predictive performance. These results revealed the necessity of regularization for accurate prediction when the covariates are large and/or some covariates are unbalancedly distributed (23).

We confirmed the clinical plausibility and applicability of the final selected predictors by expert opinion and tried to give possible explanations for the clinical findings in patients with anti-MDA5 antibodies. Previous studies work a principal component analysis (PCA)–based cluster analysis for identifying JDM subtypes, and four distinct JDM subtypes were identified (24). Among them, cluster A is characterized by the prominent symptoms of active inflammation, including higher WBCs in peripheral blood, fever, and arthralgia; it was also characterized with the highest positive rate of anti-MDA5 in this cluster, which is in accordance with the final selected predictors. It is worth noting that the symptoms of the respiratory system were not involved in our model for related predictors for the following two reasons: (1) these symptoms would likely change in a short period of time and are hard to measure, such as cough and dyspenia, which might lead to a paradoxical observation, and (2) although these symptoms are considered closely related to ILD, the lack of specificity and statistical significance during comparison between the two groups made them fail to be selected by LASSO regression analysis. Radiological examination was chosen to confirm the diagnosis of ILD instead of these symptoms, although it may lead to additional potential risks that were regarded as unnecessary for screening anti-MDA5 antibodies. The 12 selected predictors are representative and covered diverse organs typically involved in JDM, and, together, they presented a superior predictive performance.

Other certain regularization methods for high-dimensional regression perform better than LASSO. Examples are the elastic net (25), sparse Laplacian penalty (26), and so on. However, more than one tuning parameter in these models make the computation more difficult and complicated. Selecting multiple tuning parameters and applying them to the clinical application would be interesting and challenging in our future work.

There are also some limitations in our study. Anti-MDA5 positive JDM is a relatively rare subtype of JDM, and the delay between the initial diagnosis and the detection of anti-MDA5 may last for several months or years. The results of this study should then be confirmed by an external validation dataset, and the generalizability of the results, especially to adults or other regions and races, should be carefully evaluated. More research work is necessary to confirm our proposed models. Furthermore, as a cross-sectional study, patients who were not detected positive for anti-MDA5 may be excluded from this study. Therefore, the applicability of the model for patients with JDM under specific conditions (e.g., in a specific period and due to technological or economical reasons) might be limited.

In conclusion, we established an internally validated screening model for anti-MDA5 with favorable effectiveness in JDM patients from a single-center cohort using easily accessible clinical data. Though external validation will be required to demonstrate the accuracy of this model in different conditions, it would still be significant in screening anti-MDA5 antibodies in certain patients.

## Data availability statement

The original contributions presented in the study are included in the article/**Supplementary Material**. Further inquiries can be directed to the corresponding author.

## Ethics statement

The studies involving human participants were reviewed and approved by the medical ethics committee of Beijing Children’s Hospital, Capital Medical University (IEC-C-028-A10-V.05). Written informed consent to participate in this study was provided by the participants’ legal guardian/next of kin. Written informed consent was obtained from the minor(s)’ legal guardian/next of kin for the publication of any potentially identifiable images or data included in this article.

## Author contributions

CFL initiated the investigation. YX and JD wrote the manuscript. YX, SL, XT, and CL analyzed the data. JZ and WK contributed to data collection and reviewed the manuscript. All authors gave the final approval of submitting and publishing this manuscript. Study design: CFL; JZ, and YX; Data collection: JZ; Statistical analysis: YX and JZ; Data interpretation: YX and JZ; Manuscript preparation: CFL, JZ, YX, WK, and SL, Literature search: CL, XT, and JW. All authors contributed to the article and approved the submitted version.
