# Supplementary material for: Machine learning for screening and predicting the risk of anti-MDA5 antibody in juvenile dermatomyositis children
Source: Front Immunol. 2023 Jan 10;13:940802. doi: 10.3389/fimmu.2022.940802 (PMC9872019; doi:10.3389/fimmu.2022.940802)
Supplement: Supplementary file 1 [file DataSheet_1.pdf]

SUPPLEMENTARY METHODS

Selection of candidate variables

Clinical data from baseline evaluation was performed with the medical records in our center.The original variables were selected with the following details: 1.frequently detected and available in JDM;2. no large-scale missing data; 3. variables with similar significance were combined, such as arthritis and arthralgia, variable for further analysis. Each variable was measured by a rheumatologist separately to determine the stability and predictive value. Literature review was also performed to identify variables with sufficient evidence as predictors in the model. In the end, 19 potential candidate variables were considered for inclusion in the prediction model. We aimed to select the typical manifestations with specificity covering each system, and the variables with similar significance were pre-processed.The selected 19 candidate variables were shown in Table 1.

Variable selection using the least absolute shrinkage and selection operator (LASSO) logistic poisson model

Variable selection using the least absolute shrinkage and selection operator (LASSO) logistic poisson model was conducted. LASSO model coefficient profiles of the 19 candidate variables were shown in Figure 2.The optimal values on the basis of the minimum criteria and 1−SE criteria were shown in Figure 3.A λ value of 0.04194893, with a log(λ) value of −4.356293, was chosen according to cross-validation.With that λ value, a preliminary model including 12 selected varibales shown in Table 2 was established for following validation.

SUPPLEMENTARY TABLES

When no better explanation for the symptoms and signs exists, these classification criteria can be used			
	Score points		
Variable	Without muscle biopsy	With muscle biopsy	Definition
**Age of onset**			
Age of onset of first symptom assumed to be related to the disease ≥18 years and <40 years	1.3	1.5	18 ≤ age (years) at onset of first symptom assumed to be related to the disease <40
Age of onset of first symptom assumed to be related to the disease ≥40 years	2.1	2.2	Age (years) at onset of first symptom assumed to be related to the disease ≥40
**Muscle weakness**			
Objective symmetric weakness, usually progressive, of the proximal upper extremities	0.7	0.7	Weakness of proximal upper extremities as defined by manual muscle testing or other objective strength testing, which is present on both sides and is usually progressive over time
Objective symmetric weakness, usually progressive, of the proximal lower extremities	0.8	0.5	Weakness of proximal lower extremities as defined by manual muscle testing or other objective strength testing, which is present on both sides and is usually progressive over time
Neck flexors are relatively weaker than neck extensors	1.9	1.6	Muscle grades for neck flexors are relatively lower than neck extensors as defined by manual muscle testing or other objective strength testing
In the legs, proximal muscles are relatively weaker than distal muscles	0.9	1.2	Muscle grades for proximal muscles in the legs are relatively lower than distal muscles in the legs as defined by manual muscle testing or other objective strength testing
**Skin manifestations**			
Heliotrope rash	3.1	3.2	Purple, lilac-colored, or erythematous patches over the eyelids or in a periorbital distribution, often associated with periorbital edema
Gottron's papules	2.1	2.7	Erythematous to violaceous papules over the extensor surfaces of joints, which are sometimes scaly. May occur over the finger joints, elbows, knees, malleoli, and toes
Gottron's sign	3.3	3.7	Erythematous to violaceous macules over the extensor surfaces of joints, which are not palpable
**Other clinical manifestations**			
Dysphagia or esophageal dysmotility	0.7	0.6	Difficulty in swallowing or objective evidence of abnormal motility of the esophagus
**Laboratory measurements**			
Anti–Jo-1 (anti–histidyl–transfer RNA synthetase) autoantibody present	3.9	3.8	Autoantibody testing in serum performed with standardized and validated test, showing positive result
Elevated serum levels of creatine kinase (CK)* *or* lactate dehydrogenase (LDH)* *or* aspartate aminotransferase (ASAT/AST/SGOT)* *or* alanine aminotransferase (ALAT/ALT/SGPT)*	1.3	1.4	The most abnormal test values during the disease course (highest absolute level of enzyme) above the relevant upper limit of normal
**Muscle biopsy features—presence of:**			
Endomysial infiltration of mononuclear cells surrounding, but not invading, myofibers		1.7	Muscle biopsy reveals endomysial mononuclear cells abutting the sarcolemma of otherwise healthy, non-necrotic muscle fibers, but there is no clear invasion of the muscle fibers
Perimysial and/or perivascular infiltration of mononuclear cells		1.2	Mononuclear cells are located in the perimysium and/or located around blood vessels (in either perimysial or endomysial vessels)
Perifascicular atrophy		1.9	Muscle biopsy reveals several rows of muscle fibers, which are smaller in the perifascicular region than fibers more centrally located
Rimmed vacuoles		3.1	Rimmed vacuoles are bluish by hematoxylin and eosin staining and reddish by modified Gomori trichrome stain

* Serum levels above the upper limit of normal.

Supplementary Table1. 2017EULAR/ACR classification criterion for dermatomyositis

No.	Mi-2	TIF1-γ	MDA5	NXP2	SAE1	Ku	PM-Scl100	PM-Scl75	Jo-1	SRP	PL-7	PL-12	EJ	OJ	Ro-52	NO.	Mi-2	TIF1-γ	MDA5	NXP2	SAE1	Ku	PM-Scl100	PM-Scl75	Jo-1	SRP	PL-7	PL-12	EJ	OJ	Ro-52
1	-	-	-	-	-	-	-	-	-	-	-	-	-	-	-	25	-	-	-	-	-	-	-	-	-	-	-	-	-	-	+
2	-	-	+	-	-	-	-	+	-	-	-	-	-	-	-	26	+	-	++	-	-	-	-	-	-	-	-	-	-	-	-
3	+	-	-	-	-	+	-	-	-	+	-	-	-	-	-	27	-	-	-	+++	-	-	-	-	-	-	-	-	-	-	+
4	-	+	+	-	-	-	-	-	-	-	+	-	-	-	+	28	-	-	++	-	-	-	-	-	-	-	-	-	-	-	+
5	-	-	++	-	-	-	-	-	-	-	-	-	-	-	+	29	-	-	+	-	-	-	-	-	-	-	-	-	-	-	+
6	-	-	-	+++	-	-	-	-	-	-	-	-	-	-	-	30	-	-	-	-	-	-	-	-	-	-	-	-	-	-	+
7	-	-	-	-	-	-	-	-	-	-	-	-	-	-	-	31	-	-	-	++	-	-	-	-	-	-	-	-	-	-	+
8	-	-	++	+++	-	-	-	-	-	-	-	-	-	-	-	32	-	-	-	-	-	-	-	-	+	-	-	-	-	-	-
9	-	-	-	-	-	-	+	-	-	-	-	-	-	-	+	33	-	-	-	+	-	-	-	-	-	-	-	-	-	-	-
10	-	-	-	++	-	-	-	-	-	-	-	-	-	-	-	34	-	-	-	-	-	-	-	+	-	-	-	-	-	-	+
11	-	+	-	+	-	-	-	-	-	-	-	-	-	-	+	35	-	-	+	-	-	-	-	-	-	-	-	-	-	-	+
12	-	-	++	-	-	-	-	-	-	-	-	-	-	+	-	36	-	-	-	-	-	-	-	-	-	-	-	-	-	-	-
13	-	-	-	-	-	-	-	-	-	-	-	-	-	-	-	37	-	+	-	-	-	-	-	-	-	-	-	-	-	-	-
14	-	-	-	-	-	-	-	-	-	-	-	-	-	-	-	38	+	-	-	+	-	-	-	-	-	-	+	-	-	-	-
15	-	-	+++	++	-	-	-	-	-	-	-	-	-	-	+++	39	-	-	+++	-	-	-	-	-	-	-	-	-	-	-	-
16	-	-	-	-	-	-	-	-	-	-	-	-	-	-	-	40	-	-	-	++	-	-	-	-	-	-	-	-	-	-	-
17	-	-	-	++	-	-	-	-	-	-	-	-	-	-	-	41	-	-	+	-	-	-	-	-	-	-	-	-	-	-	-
18	-	-	+	-	-	-	-	-	-	-	-	-	-	-	-	42	+	-	-	-	-	-	-	-	-	-	-	-	-	-	-
19	-	-	++	-	-	-	-	-	-	-	-	-	-	-	+	43	+	-	+	-	-	-	-	-	-	-	-	-	-	-	-
20	-	-	-	-	-	-	-	-	-	-	-	-	-	-	-	44	-	-	-	-	-	-	-	-	-	-	-	-	-	-	-
21	-	-	-	+++	-	-	-	-	-	-	-	-	-	-	-	45	-	+	-	-	-	-	-	+	-	-	-	-	-	-	-
22	-	-	-	+	-	-	-	-	-	-	-	-	-	-	-	46	-	++	-	-	-	-	-	-	-	-	-	-	-	-	+
23	-	-	-	-	-	-	-	-	-	-	-	-	-	-	-	47	+	-	-	-	-	-	-	-	-	-	-	-	-	-	+

24	-	-	-	-	-	-	-	-	-	-	-	-	-	-	-	48	-	-	+++	-	-	-	-	-	+	-	-	-	-	-	-
49	-	-	-	-	-	-	-	-	-	-	-	-	-	-	-	77	-	-	-	+++	-	-	-	-	-	-	-	-	-	-	+
50	-	-	+	-	-	-	-	-	+	-	-	-	-	-	-	78	-	-	-	-	-	-	-	-	-	-	-	-	-	-	-
51	-	-	-	-	-	-	-	-	-	-	-	-	-	-	-	79	-	-	-	++	-	-	-	-	-	-	-	+	-	-	-
52	-	-	++	-	-	-	-	-	-	-	-	-	-	-	-	80	-	-	-	+	-	-	-	-	-	-	-	-	-	-	-
53	-	-	-	-	-	-	-	-	-	-	-	-	-	-	-	81	-	+	++	-	-	-	-	-	-	+	-	-	-	-	-
54	-	-	+	-	-	-	-	-	-	-	-	-	-	-	+	82	-	-	-	-	-	-	-	-	-	-	-	-	-	-	-
55	-	-	-	+++	-	-	-	-	-	-	-	-	-	-	+	83	-	-	-	-	-	-	-	-	-	+	+	+	-	-	+
56	-	-	+++	+++	-	-	-	-	-	-	-	-	-	-	+	84	-	-	++	-	-	-	+	-	-	-	-	-	-	-	+
57	-	+	-	-	-	-	-	-	-	-	-	-	-	-	-	85	+	-	-	-	-	-	-	-	-	-	-	-	-	-	-
58	-	-	-	-	-	-	-	-	-	-	-	-	-	-	-	86	-	-	-	-	-	-	-	+	-	-	-	-	-	-	-
59	-	-	+	-	-	-	-	-	-	-	-	-	-	-	-	87	-	+	-	++	-	-	-	-	-	-	-	-	-	-	-
60	-	-	-	-	-	-	-	-	-	-	-	-	-	-	-	88	-	-	+	-	-	-	-	-	-	-	-	-	-	-	-
61	-	+	-	-	-	-	-	-	-	-	-	-	-	-	+	89	-	-	+	-	-	-	-	-	-	-	-	-	-	-	-
62	-	+	++	-	-	-	-	-	-	-	-	-	-	-	+	90	-	-	-	-	-	-	-	-	-	-	-	-	-	-	-
63	-	-	-	+++	-	-	-	-	-	-	-	-	-	-	+	91	-	-	-	-	-	-	-	-	-	-	-	-	-	-	-
64	-	-	-	+++	-	-	-	-	-	-	-	-	-	-	++	92	-	-	-	-	-	-	-	-	-	-	-	-	-	-	-
65	-	-	-	-	-	-	-	-	-	-	-	-	-	-	-	93	+	+	++	-	-	+	-	-	-	-	-	-	-	-	++
66	-	-	+	-	-	-	-	-	-	-	-	-	-	+	+	94	-	-	-	++	-	-	-	+	-	-	-	-	-	-	+
67	-	-	-	-	-	-	-	-	-	-	-	-	-	-	-	95	+	+	++	-	-	-	-	-	-	-	-	-	-	-	+
68	-	-	-	-	-	-	-	-	-	-	-	-	-	-	-	96	-	+++	-	-	-	-	-	-	-	-	-	-	-	-	-
69	+	+	-	-	-	-	-	-	-	-	-	-	-	-	-	97	+	+	-	-	-	-	-	-	-	-	-	-	-	-	-
70	-	-	-	-	-	+	-	-	-	-	-	-	-	-	-	98	-	-	-	-	-	+	+	-	-	-	-	-	-	-	-
71	+	-	+++	-	-	-	-	+	-	+	-	-	-	-	-	99	-	+++	+	-	-	-	-	+++	-	++	-	-	-	-	-
72	-	-	-	+	-	-	-	-	-	-	-	-	-	-	-	100	+	-	-	+++	-	-	-	-	-	-	-	-	-	-	+
73	-	-	-	-	-	-	-	-	-	-	-	-	-	-	-	101	+	-	-	-	-	-	-	-	-	-	-	-	-	-	-

74	-	-	-	-	-	-	-	-	-	-	-	-	-	-	-
75	-	-	+	-	-	+	+	-	-	-	-	-	-	-	-
76	-	-	-	-	-	-	+	-	-	-	-	-	-	+	+
102	-	++	+	-	-	-	-	-	-	-	-	-	-	-	+
103	-	-	++	-	-	-	-	+	-	+	-	-	-	-	-
104	-	++	-	-	-	-	-	-	-	-	-	-	-	-	+
105	+	-	-	-	-	-	-	-	-	-	-	-	-	-	+
106	+	-	++	+++	-	-	-	-	-	-	-	-	-	-	-
107	-	+++	-	-	-	-	-	-	-	-	-	-	-	-	+
108	+	-	+	-	-	-	-	-	-	-	-	-	-	-	-
109	-	-	-	-	-	-	-	+	-	-	-	-	-	-	+
110	-	-	+	+++	-	-	-	-	-	-	-	-	-	-	-
111	-	-	-	-	-	-	-	-	-	-	-	-	-	-	+
112	-	+	-	-	-	-	-	-	-	-	-	-	-	-	+
113	-	-	++	-	-	-	-	-	-	-	-	-	-	-	+
114	-	+	-	-	-	+	-	-	-	-	-	-	-	-	-
115	-	+	-	+	-	-	-	-	-	-	-	-	-	-	+
116	-	+	-	+	-	-	-	-	-	-	-	-	-	-	-
117	-	-	-	-	-	-	-	-	-	-	-	-	-	-	-
118	-	-	-	-	-	-	+	-	-	-	-	-	-	+	-
119	-	-	-	-	-	-	+	-	-	-	-	-	-	-	-
120	-	-	-	-	-	-	-	-	-	-	-	-	-	-	-
121	-	+	-	-	-	-	-	-	-	-	-	-	-	-	-
122	-	-	-	-	-	-	-	-	-	-	-	-	-	-	-
123	-	-	-	-	-	-	-	-	-	-	-	-	-	-	-
124	-	-	-	-	-	-	-	-	-	-	-	-	-	-	-
125	-	+	-	-	-	-	-	-	-	-	-	-	-	-	-
126	+	+	-	+	+	+	-	-	-	+	-	+	-	+	-
127	-	+	-	+	-	-	-	-	-	-	-	-	-	-	+

128	-	+	-	+	-	-	-	-	-	-	-	-	-	-	-
129	-	-	-	-	-	-	-	-	-	-	-	-	-	-	-
130	-	-	-	-	-	-	+	-	-	-	-	-	-	+	-
131	-	-	-	-	-	-	+	-	-	-	-	-	-	-	-
132	-	-	-	-	-	-	-	-	-	-	-	-	-	-	-
133	-	+	-	-	-	-	-	-	-	-	-	-	-	-	-
134	-	-	-	-	-	-	-	-	-	-	-	-	-	-	-
135	-	-	-	-	-	-	-	-	-	-	-	-	-	-	-
136	-	-	-	-	-	-	-	-	-	-	-	-	-	-	-
137	-	+	-	-	-	-	-	-	-	-	-	-	-	-	-
138	+	+	-	+	+	+	-	-	-	+	-	+	-	+	-
139	-	-	-	-	-	+	-	-	-	+	-	-	-	-	-
140	-	+	-	-	-	-	-	-	-	-	-	-	-	-	+
141	-	-	-	-	-	-	-	-	-	-	-	-	-	-	+
142	-	-	-	+++	-	-	-	-	-	-	-	-	-	-	+
143	-	-	-	-	-	-	-	-	-	-	-	-	-	-	+
144	-	-	-	-	-	-	+	-	-	-	-	-	-	-	-
145	-	-	-	-	-	-	-	-	-	-	-	-	-	-	-
146	-	+	-	-	-	-	-	-	-	-	-	-	-	-	-
147	-	-	-	-	-	-	-	-	-	-	-	-	-	-	-
148	-	-	-	-	-	-	-	-	-	-	-	-	-	-	-
149	-	-	-	-	-	-	-	-	-	-	-	-	-	-	-
150	-	+	-	-	-	-	-	-	-	-	-	-	-	-	-
151	+	+	-	+	+	+	-	-	-	+	-	+	-	+	-
152	-	-	-	-	-	+	-	-	-	+	-	-	-	-	-

Supplementary Table2 .The distribution of the 152 patients' myositis associated antibodies.
